# The Development of a New Shock Absorbing Uniaxial Graded Auxetic Damper (UGAD)

**DOI:** 10.3390/ma12162573

**Published:** 2019-08-12

**Authors:** Hasan Al-Rifaie, Wojciech Sumelka

**Affiliations:** Institute of Structural Engineering, Poznan University of Technology, 60-965 Poznan, Poland

**Keywords:** damper, auxetic material, extreme loading, blast, impact

## Abstract

Auxetic structures are efficient cellular materials that can absorb blast/impact energy through plastic deformation, thus protecting the structure. They are developing sacrificial solutions with light weight, high specific strength, high specific toughness and excellent energy dissipating properties, due to its negative Poison’s ratio nature. The use of auxetic and non-auxetic panels in blast resistant structures had been relatively perceived by researchers. Nonetheless, implementation of those energy dissipaters, explicitly as a uni-axial passive damper is restrained to limited studies, which highlight the potential need for further explorations. The aim of this paper is the design of a new uniaxial graded auxetic damper (UGAD) that can be used as a blast/impact/shock absorber in different scales for different structural applications. First, the geometry, material, numerical model and loading are introduced. Then, a detailed parametric study is conducted to achieve the most efficient graded auxetic system. Moreover, the designed auxetic damper is numerically tested and its static and dynamic constitutive relations are derived and validated analytically. The selection of optimum parameters was based on the ratio of the reaction force to the applied load (RFd/P) and plastic dissipation energy (PDE). The final designed UGAD contains three auxetic cores that have the same geometry, material grade (6063-T4), size and number of layers equal to eight. The cell-wall thickness ***t*** of the three auxetic cores is 1.4 mm, 1.8 mm and 2.2 mm, respectively; composing a graded auxetic system. The performance of the three auxetic cores together have led to a wide plateau region (80% of total crushing strain) and variant strength range (1–10 MPa), which in return, can justify the superior performance of the UGAD under different blast levels. Finally, the 3D printed prototype of the UGAD is presented and the possible applications are covered.

## 1. Introduction 

Damping can be defined as the phenomenon by which mechanical energy is dissipated in dynamic systems [1]. When a structure does not have enough damping to absorb a required level of dynamic loading, additional external dampers are needed. Dampers are devices that dissipate energy through some sort of motion. They are used in mechanical, civil and aerospace applications [2]. In multi-storey buildings, as an example, damping systems are extensively used as seismic vibration controllers [3,4,5]. Dampers are either passive (works without external power need) or active (have actuators and sensors that require external power). One of the extreme damping systems’ applications is connected with the dissipating blast wave. In such a case, the “passive” type of dampers is more favoured as an external power cut is most probable. In return, such energy absorbing systems protect human lives and properties [6]. After 9/11, the US department of Defence and Homeland Security urged the need for high capacity blast absorbers [6]. In addition, Monir [7] states that the attenuation of blast effects using passive yielding dampers is a complicated subject that requires more investigation [7].

Cellular materials; such as metal foams, honeycomb and auxetics; are developing alternatives with light weight, high specific strength, high specific toughness and good energy dissipating properties [8,9]. They are generally in the form of sacrificial sandwich panels that absorbs blast energy through plastic deformation, thus protecting the structure. In auxetics, the density increases more rapidly with lateral contraction. Consequently, the energy absorption capacity can be specified as the energy required to deform a specimen to its full densification strain. Despite the potential use of aluminium foams in blast and impact applications, the irregularity in its microstructure makes it difficult to optimize foam properties to the applied load. Peroni et al. [10] state that primary issues in the analysis of aluminium foams are large density scatter and material anisotropy. In return, problems could arise in the evaluation of mechanical properties for real applications. To tackle this barrier, honeycomb and auxetic structures are promising solutions. Honeycomb structures are used in a wide range of shock absorption applications due to their impact resistant and energy absorption characteristics [11,12,13,14]. Analytical [15,16], numerical [17,18] and experimental [19,20] studies have been conducted to describe their mechanical properties and response. However, recent studies confirm that the unique behaviour of the negative Poisson’s ratio in auxetic structures provides better energy absorption than the traditional honeycomb (hexagonal) topology [21,22]. Hence, auxetic structures were selected for the design of the passive damping system in this paper. Recent advances in auxetic structures and comparisons with the honeycomb performance are provided in the following sections in detail.

Auxetics are defined as solids that possess the negative Poisson’s ratio [23]. The negative Poisson’s ratio (or auxetic behaviour) means that when an auxetic sample is stretched in one direction, it expands in the other direction. Reversely, when it is compressed, it contracts in the transverse direction [22,24]. A number of review studies concerning auxetic materials/structures and their application were conducted. These include, but not limited to, the research of Lakes [25], Alderson [26], Yang, et al. [27], Alderson and Alderson [28], Liu and Hu [29], Greaves [30] and Prawoto [31].

The auxetic nature in a body originates either naturally (from the material itself) or artificially (changing the geometry on the micro-structure level). Naturally occurring materials that exhibit the negative Poisson’s ratio, such as α-cristobalite silicon dioxide [32], are rarely used in engineering applications. The more common is the geometry-related auxetic nature. Examples of cellular geometries, that give auxetic behaviour, are double arrow-head [33], re-entrant [34], chiral [35], and rotating rigid units [36]. They are used to produce foams or auxetic cellular metals, for a wide range of applications, such as aerospace, biomedical and military engineering [37]. 

Researchers have demonstrated that auxetic foams show higher strength to weight ratio, lower stiffness and better energy absorption than conventional ones [25,38,39,40,41,42]. In addition, sandwich panels with auxetic cores; have been investigated under static and blast-induced dynamic shockwaves. Enhanced damage localization [43,44], flexural response [45], indentation resistance [46,47,48], and energy absorption [49,50,51], were obtained. It is worth mentioning that three-dimensional auxetic structures have also been developed [52]. They have a form of multipod lattice [53], an auxetic frame [54] or bow-tie elements [55]. However, in order to manufacture a 3D auxetic structure, sophisticated processes are required accompanied by different challenges due to technological limitations [52,56]. On the other hand, 2D auxetics can be manufactured through profile-rolling of sheet-metal blanks [51], slotting metal sheets [57] or by 3D printing [58,59,60]. Based on the remarks above, the 2D re-entrant topology was implemented in this paper, due to its relatively-simple geometry, and less expensive fabrication, compared to other auxetic topologies. In addition, research in this field show that the abilities of the re-entrant auxetic topology are still waiting to be uncovered, tested and verified [49]. The analysis of dynamic crushing of cellular materials is efficiently performed through numerical FEA tools [61,62], as experimental approaches need enormous resources [49]. In literature, better performance of re-entrant auxetics is assessed based on comparisons with the nonauxetic hexagonal honeycomb of the same properties. The simple geometries of the Auxetics and nonauxetic hexagonal honeycomb allow a direct optimization process to their blast absorption capabilities through modifying their geometrical parameters. 

Some more information may be useful to the reader here; explaining why being auxetic improves strength, toughness and energy dissipation. Greaves et al. [63], cover auxetic’s crack propagation, as discussed in his review paper “Poisson’s ratio and modern materials”. Wenwang et al. [64] mention that auxetic structures exhibit enhanced mechanical properties compared to conventional materials, ranging from shearing modulus, increased indentation resistance and higher fracture toughness, thus serving a wide range of industrial applications. In Alderson et al. [65], the low velocity impact response of auxetic carbon fibre laminates was compared to the response of non-auxetic equivalent laminates. The auxetic laminates showed better energy absorption and smaller damage area. Enhancements in the indentation resistance were also confirmed by Coenen and Alderson [66] for the auxetic carbon fibre laminates, with smaller, more localized damage areas.

In short, to the author’s knowledge, the use of auxetic and non-auxetic dampers in blast resistant structures had been relatively perceived by researchers. Nonetheless, implementation of those energy dissipaters, explicitly as a uni-axial passive damper is restrained to limited studies, which highlight the potential need for further explorations. The aim of this paper is the (design, elaboration and assessment) of a new uniaxial graded auxetic damper (UGAD). First, the geometry, material, numerical model and loading are introduced. Then, a detailed parametric study is conducted to achieve the most efficient graded auxetic system. Moreover, the designed auxetic damper is tested and its static and dynamic constitutive relations are derived and validated analytically. Finally, the possible applications of this system are covered.

## 2. General Concept of the UGAD

**Figure 1:**
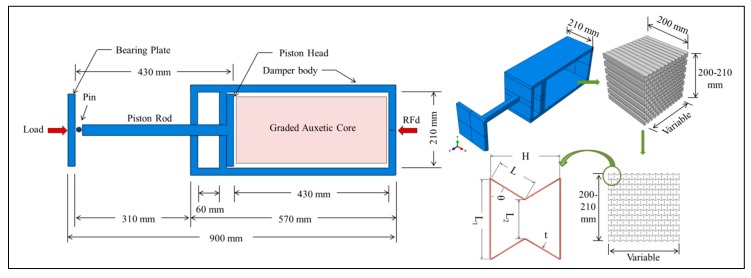
Geometry and components of the proposed uniaxial graded auxetic damper (UGAD).

The uniaxial graded auxetic damper (UGAD) proposed in the research consists of four main components, which are the bearing plate, piston, damper body and the graded auxetic core (Figure 1). The bearing plate has 200 × 200 × 10 mm dimensions and is the first damper component that receives the impact load. The bearing plate is pinned to the piston rod that transfers the load to the piston head. The piston is sliding inside the damper body compressing the auxetic core. The core is supposed to be a relatively cheap sacrificial auxetic structure that can be easily changed after a blast or impact event. The auxetic core main task is absorbing the impact energy and reducing reaction forces on whole system supports. The damper reaction force will be denoted here as (RFd). 

The overall length of the damper is 900 mm (uncompressed) and 590 mm (fully compressed). The damper body internal chamber has clear dimensions of 210 × 210 × 430 mm, where the auxetic core is situated. The focus, in this study, is on the parametric design of the auxetic core, rather than other components (damper body, piston or bearing plates), as those components are more stiffwith negligible energy absorption characteristic. Figure 1 shows the geometry of UGAD with dimensions of all its components.

In terms of the auxetic core, Table 1 lists fixed and variable geometrical parameters that play a crucial role in the overall design process of the auxetic core. The extrusion depth and height of the core are specified while the length is variable. Based on the parametric study conducted in Section 4, optimum cell dimensions, cell angle, number of layers, material and thickness t, will be selected.

## 3. Modelling Techniques and Assumptions 

### 3.1. Numerical Model 

**Figure 2:**
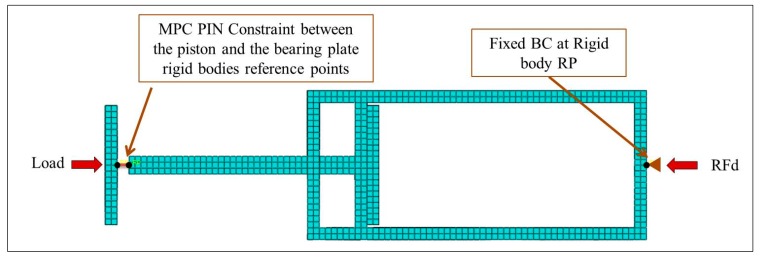
**Boundary conditions** and constraints of the bearing plate, piston and damper body.

Simulia Abaqus (version 2016) is the computational tool that has been used in this study, implementing the explicit solver. As mentioned earlier, the design of the bearing plate, piston and damper body are out of the scope of this research. Therefore, they were modelled as 3D parts with rigid body constraints applied to each one of them separately. Boundary conditions and loading were applied to their reference points (RP) as shown in Figure 2. They were meshed using the C3D8R element type (an eight node linear brick, reduced integration) with a mesh size of 10 mm.

**Figure 3:**
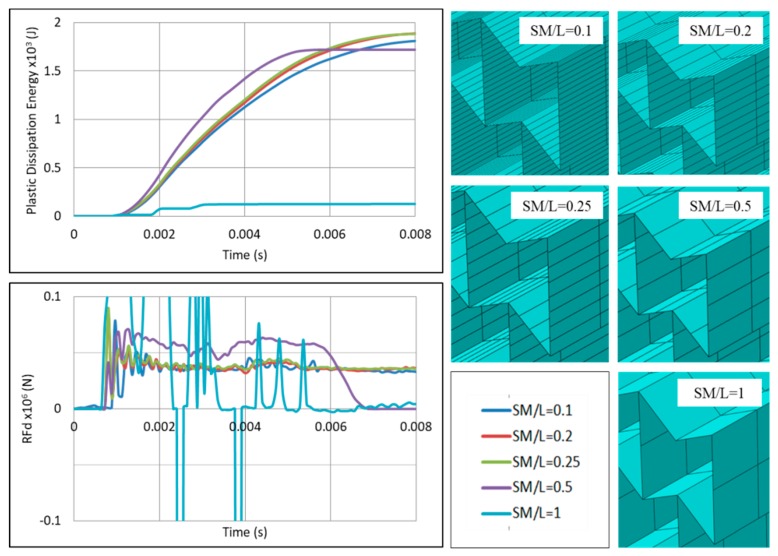
Mesh sensitivity study. Finding the most accurate-less expensive auxetic core model (different SM/L ratios), based on comparing plastic dissipation energy (PDE) and reaction force (RFd), for an auxetic core of L = 10 mm, t = 1 mm, S4R elements, AL3 aluminium, pulse load of 0.5 × 10^6^ N in 0.002 s.

In terms of the auxetic core, the mesh element type was S4R, which is a four-node doubly curved shell with reduced integration. A detailed quantitative mesh analysis was conducted to find the more accurate-less expensive (cost-based) element size (Figure 3). The analysis was for certain auxetic core parameters and loading condition with changing the size of the mesh (denoted here as SM). It was found that the more accurate-less expensive element size was when SM/L = 0.25 (i.e., when the mesh size is quarter the cell wall length L). Additional analysis for the mesh size in the extrusion direction found to have no effect on results, as the auxetic core is uniform in the extrusion direction. Hence, it was set equal to the value of L.

### 3.2. Constitutive Law for The Auxetic Core 

An elasto-plastic model with damage initiation was used for the dynamic simulations of the UGAD. Plasticity and damage were defined using the Johnson-Cook model. 

The Johnson-Cook material model is one of the semi-empirical constitutive models that can describe the plastic material behaviour at high strains, high strain rates and high temperatures. The model (in Equation (1)) describes the yield stress $\sigma_{y}$ and takes into account the strain rate hardening and thermal softening effects [67,68,69,70]. The dimensionless temperature parameter $\hat{T}$ is defined in Equation (2).

(1) $$\sigma_{y}=\left(A+B\;\varepsilon^{n}\right)\;\left[1+C\;\ln\left(\frac{\dot{\varepsilon }}{\dot{\varepsilon_{0}}}\right)\right]\;\left[1-{\left(\hat{T}\right)}^{m}\right]$$

(2) $$\hat{T}=0\;\mathrm{for}\;T<T_{0}\;\hat{T}=\frac{T-T_{0}}{T_{m}-T_{0}}\;\mathrm{for}\;T_{0}<T<T_{m}\;\hat{T}=1\;\mathrm{for}\;T>T_{m}$$

where, ε is the plastic strain, $\dot{\varepsilon }$ is the plastic strain rate, $\dot{\varepsilon_{0}}$ is the reference plastic strain rate, T is the current material temperature, $T_{m}$ is the melting point of the material, and $T_{0}$ is the transition/room temperature at or below which there is no temperature dependence of the yield stress. *A*, *B*, *C*, *n* and *m* are material parameters measured at or below $T_{0}$. *A* is the yield stress, *B* is the pre-exponential factor, *C* is the strain rate factor, *n* is the work-hardening exponent and *m* is the thermal-softening exponent. 

In addition, the Johnson-Cook dynamic failure model is supplied by ABAQUS/Explicit, version 2016, Johnston RI, USA [71]. The failure is assumed to happen when the damage parameter ω exceeds one. The damage parameter is defined as:

(3) $$\omega =\sum \left(\frac{\Delta \varepsilon \;}{\varepsilon_{f}}\right)$$

where, Δ*ε* is an increment of the plastic strain, *ε_f_* is the plastic strain at failure, and the summation is performed over all increments in the analysis. The plastic strain at failure ε*_f_* is dependent on the nondimensional plastic strain rate $\frac{\dot{\varepsilon }}{\dot{\varepsilon_{0}}}$, pressure to the HMH stress ratio $\frac{p}{q}$, and the dimensionless temperature parameter $\hat{T}$. The strain at failure ε*_f_* can be expressed as:

(4) $$\varepsilon_{f}=\;\left[d_{1}+d_{2}\exp\left(d_{3}\;\frac{p}{q}\right)\right]\left[1+d_{4}\;\ln\left(\frac{\dot{\varepsilon }}{\dot{\varepsilon_{0}}}\right)\right]\;\left(1+d_{5}\;\hat{T}\right)$$

where $d_{1}-d_{5}$ are failure parameters. 

To account for the material grade effect on the behaviour of the auxetic core under high strain rates, three different aluminium grades were selected (Table 2). The first one was the high strength grade AL7075-T6 (denoted here as AL1). It has a yield point of 546 MPa and used in aerospace and defence applications. The second one was the 324 MPa medium strength AL6061-T6 grade (denoted here as AL2), which is used for general structural applications. The third and the last one was the low strength grade AL6063-T4 (denoted here as AL3). This type is relatively cheaper and more available than other grades. It is widely used in manufacturing doors, windows and furniture. The material parameters are listed in Table 3 for each aluminium grade. 

**Figure 4:**
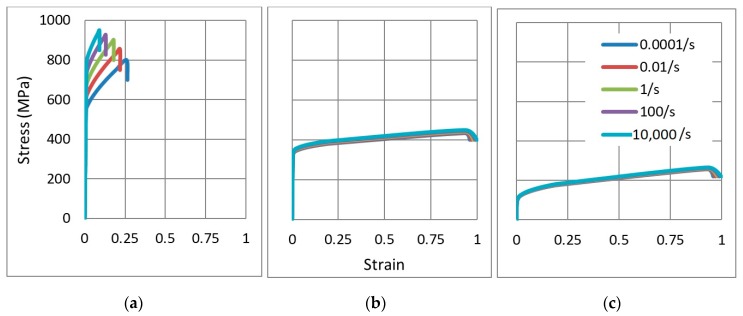
Stress-strain relationship for the three aluminium grades, at different strain rates (y-axis is the same for each subfigure), (**a**) Grade AL7075-T6 (AL1), (**b**) Grade AL6061-T6 (AL2), (**c**) Grade AL6063-T4 (AL3).

Based on Hook’s Law for the elastic range, Equation (1) for the plastic range and Equation (4) for the damage initiation point, the stress-strain curves of the three grades were drawn, for different strain rates (Figure 4). In addition, assuming $\hat{T}=0$ ($T<T_{0}$), $\frac{p}{q}=\frac{1}{3}$ for the 1D bar strain, and substituting different values of the strain rate $\dot{\varepsilon }$ and plastic strain ε, the corresponding stress and strain at failure were achieved.

According to the Stress-strain relationship for the three aluminium grades (Figure 4), AL1 can be considered as a high strength-low ductility grade that is more rate sensitive. In contrast, the other two grades show lower strength, high ductility and less rate dependency. The parametric study in Section 4.3 is dedicated for the grade used and its influence on energy absorption and reaction forces.

### 3.3. Loading 

As the main aim of the UGAD is absorbing the impact/blast energy, the loading should be based on the real-case scenario as an impulse. Therefore, the loading in this study was based on results of a previous research conducted and published by the same authors, namely “Numerical analysis of reaction forces in blast resistant gates” [75]. The study shows the extreme level of reaction forces from a blast event and the need for damping systems to mitigate them.

As Section 4 is related to the geometrical/material parametric study, the loading had to be kept the same to validate the comparisons. A pulse ‘P’ of 0.5 × 10^6^ N at 0.002 s was applied on the UGAD. The controlled parameters were the ratio of the reaction force to the applied load (RFd/P) and plastic dissipation energy (PDE). As known, solid bodies transmit applied loads directly to supports, leading to reaction forces equal to the applied load. However, auxetic structures are supposed to absorb the shock, leading to less reaction forces at the back. Therefore, the ratio RFd/P, monitored in Section 4, highlights the reduction in the reaction force, which the auxetic core may do. Results are validated and compared with the reviewed literature in Section 1.

## 4. Parametric Study 

In this section, a thorough parametric study is conducted to achieve an efficient auxetic core. The study first takes into account the loading direction on the auxetic core. Then, cell dimensions, material grades and cell angles, are checked. Lastly, the effect of the changing number of layers of the auxetic core is also covered. The study is based on changing one variable (from the mentioned above) at a time, and keeping other parameters fixed, as conducted by Imbalzano et al. [22] and Liu et al. [49].

### 4.1. Loading Direction 

As re-entrant auxetics have anisotropic properties, it’s important at first to check the direction at which the auxetic core should be loaded to achieve more PDE and less RFd. Two auxetic cores with (t = 0.75 mm, L = 5 mm, t/L = 0.15, θ = 60°, AL2 grade) were loaded in two different directions, namely here, D1 and D2. Table 4 shows the collapse and deformation modes from time 0–0.004 s for the two loading configurations.

**Figure 5:**
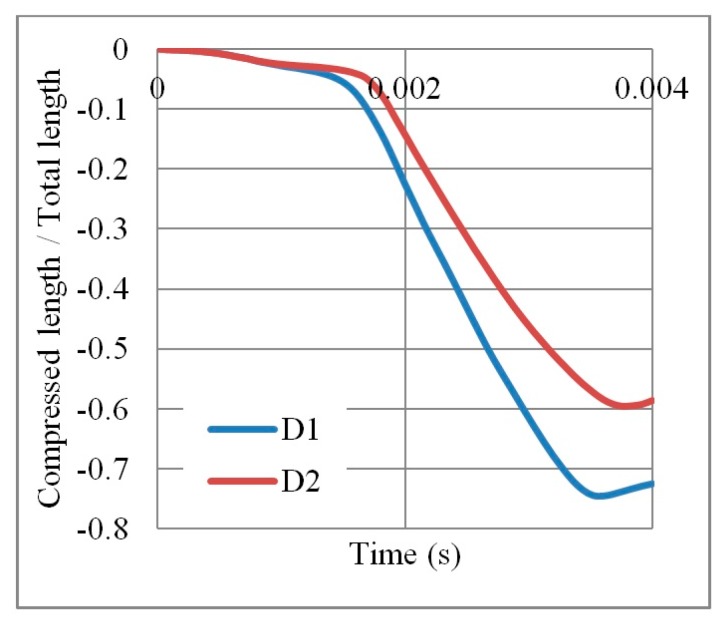
Ratio of compressed length to total length per time, for an auxetic core loaded in two different directions D1 and D2 (t  = 0.75 mm, L = 5 mm, t/L = 0.15, θ  = 60°, AL2 grade).

In terms of the loading direction D1 (Table 4), initial localization bands occur at the proximal (loaded) and distal (supported) ends that spreads quickly over the whole section. In addition, cells near the horizontal symmetry axis are compressed while those near the free boundaries are in the tension state. This leads to the transverse shrink or auxetic behaviour with Poisson’s ratio = −0.3. At the final time step, 0.004 s, the core is fully collapsed with the compressed length to total length ratio of 75% (Figure 5). 

In contrast, the loading direction D2 shows a local deformation at the proximal (loaded) end of the core, which propagates forward layer by layer to the distal (supported) end. Less necking or transverse shrink can be observed with Poisson’s ratio = −0.1. The less-auxetic behaviour of D2 for this high loading rate agrees with the findings of Zhang et al. [37]. At the final time step, 0.004 s, the core is not fully collapsed with the compressed length to total length ratio of 60% (Figure 5). 

**Figure 6:**
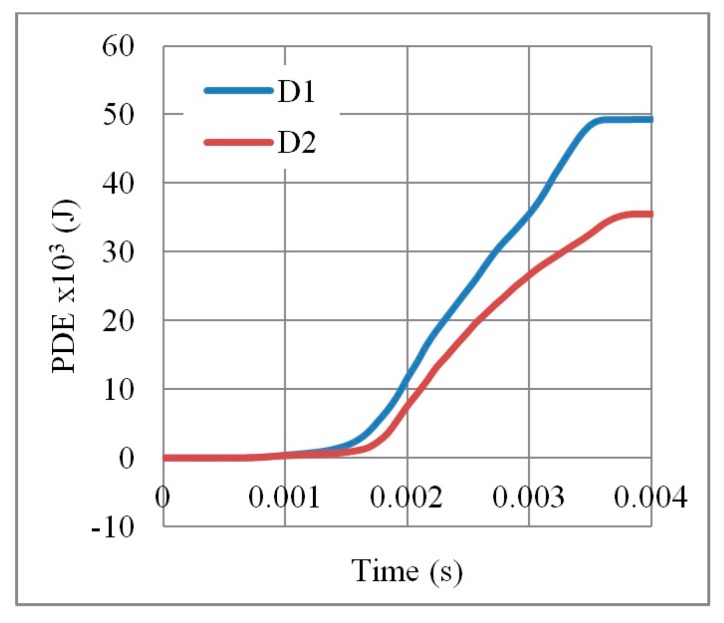
Plastic dissipation energy PDE with respect to time, for an auxetic core loaded in two different directions D1 and D2 (t  = 0.75 mm, L = 5 mm, t/L = 0.15, θ = 60°, AL2 grade).

**Figure 7:**
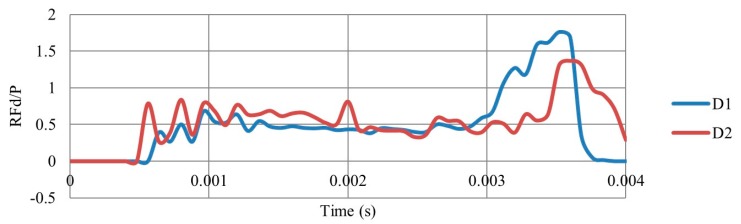
Ratio of the reaction force to the applied load (RFd/P) with respect to time, for an auxetic core loaded in two different directions D1 and D2, (t = 0.75 mm, L = 5 mm, t/L = 0.15, θ = 60°, AL2 grade). It shows that direction D1 (with higher auxetic behaviour (Table 4), gives less RFd/P.

It is evident form Figure 6 that the PDE with respect to the time of direction D1 is higher than that of D2. This can be justified to the auxetic effect that leads to more energy absorption [21]. In terms of the reaction force (Figure 7), D1 showed better performance with less RFd/P ratio, except for the final collapse reaction. The full collapse in D1 should be avoided. In short, the auxetic core would be situated in the UGAD and loaded as in direction D1 due to its better performance.

### 4.2. Cell Dimension 

Manufacturing an auxetic core with small cells is more difficult and requires precise technology compared to a core with larger cell dimensions. However, smaller cells may lead to more plastic hinges and hence more PDE. Therefore, three different auxetic cores with three different cell dimensions were tested. According to Figure 1, that shows the auxetic cell with its parameters, it is evident that L and θ are the controlling factors of the cell dimension (as L1 = 2L, and L2 relates to θ). The cell dimensions were varied here based on changing the value of L while keeping θ constant at 60°. Table 5 shows the three auxetic cores (denoted here as A, B and C) with three different cell dimensions and their properties. It was crucial also to change the wall thickness t to achieve the same t/L ratio, here fixed at 0.2.

**Figure 8:**
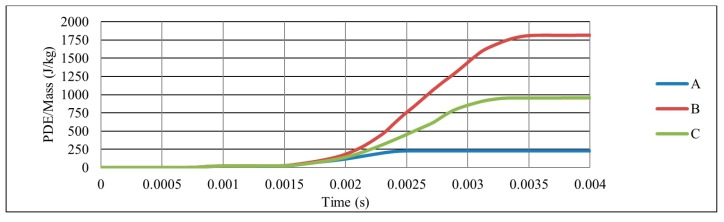
Ratio of PDE/Mass with respect to time, for three different cell dimensions A, B and C with θ = 60°, t/L = 0.2, subjected to the same loading conditions.

As the mass of the three auxetic cores were different, the PDE was divided by the mass to normalise the results. Figure 8 shows the ratio of the PDE/Mass with respect to time, for the three different cell dimensions A, B and C. It is clear that the auxetic core B (with L = 10 mm) has the best PDE among others.

**Figure 9:**
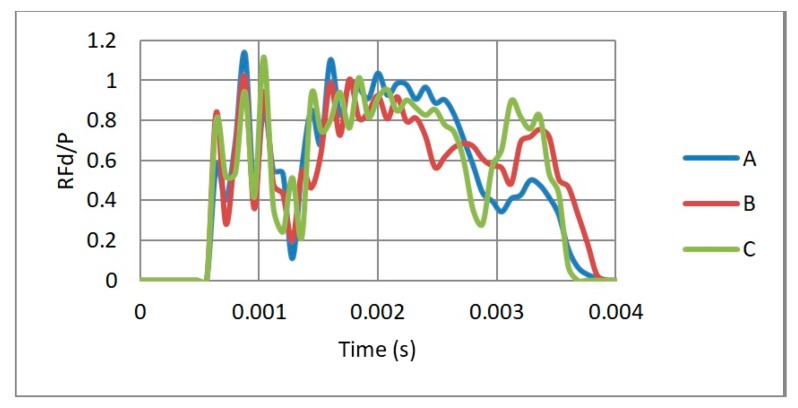
RFd/P–time history, for 3 different cell dimensions A, B and C with θ = 60°, t/L = 0.2, subjected to same loading conditions.

**Figure 10:**
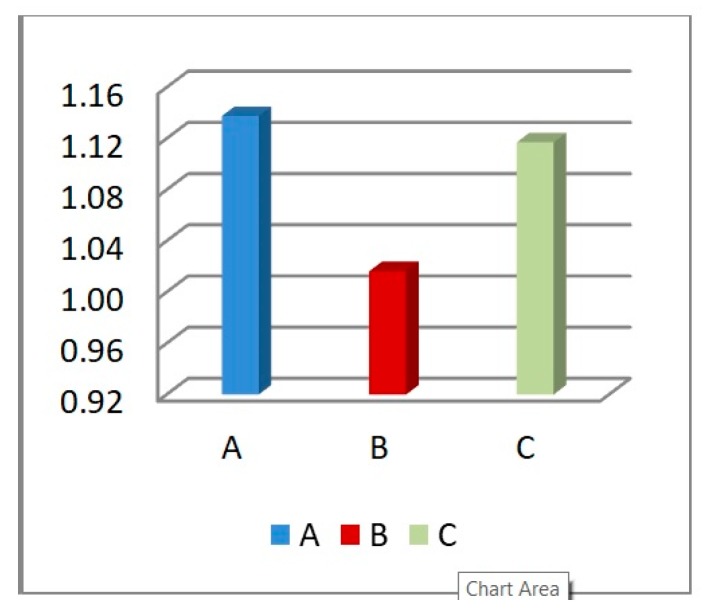
Peak value of RFd/P, for the 3 cell dimensions A, B and C.

In terms of the reaction force, Figure 9 shows the RFd/P-time history for three different cell dimensions A, B and C, while Figure 10 highlights the peak values of RFd/P. It can be noticed that the cell dimension B had the least RFd/P, leading to less reaction force, and hence stress, on the back of the damper. So cell size B, with L = 10 mm, was the selected dimension for the following sections.

### 4.3. Aluminium Grade 

The parametric study in this section is dedicated for finding the influence of the used aluminium grade on the energy absorption and reaction forces. Three aluminium grades (AL1, AL2 and AL3) were selected and described in Section 3. Three auxetic cores were tested having three different grades and same geometrical parameters (L = 10 mm, t = 2 mm, t/L = 0.2, θ = 60°) and loading condition. As the grades have different densities, and hence different mass of auxetic sections, the PDE were also normalized based on the mass. This was to validate the comparison based on the energy dissipated per each kg of material.

**Figure 11:**
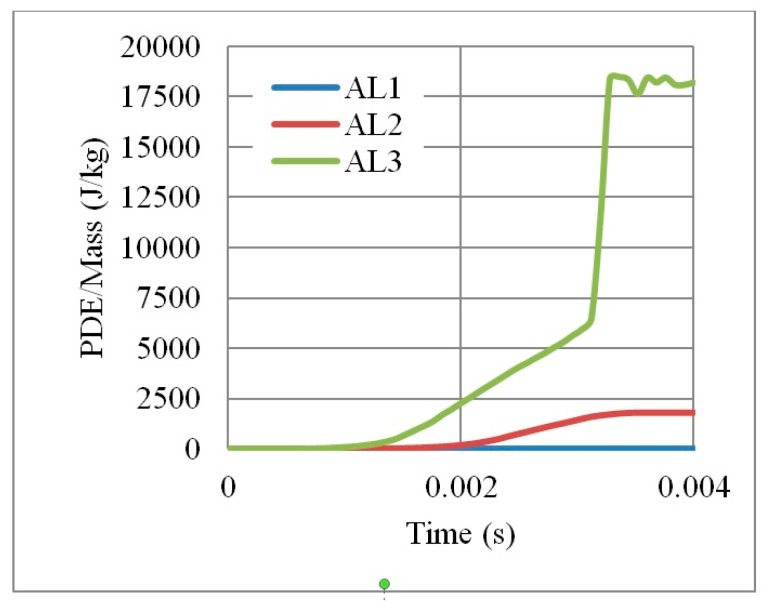
Ratio of PDE/Mass with respect to time, for 3 different Aluminium grades AL1, AL2 and AL3, of an auxetic core with L = 10 mm, t = 2mm, t/L = 0.2.

**Figure 12:**
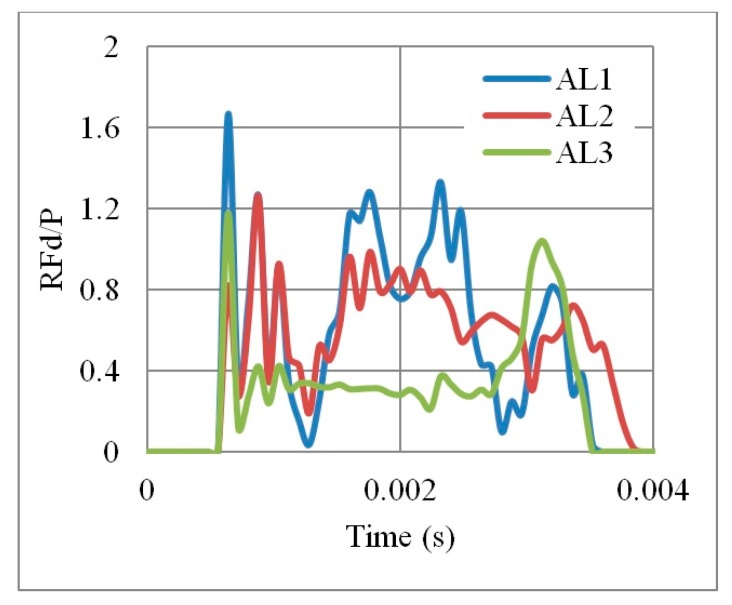
RFd/P time history, for 3 different Aluminium grades AL1, AL2 and AL3, of an auxetic core with L = 10 mm, t = 2mm, t/L = 0.2.

Results (Figure 11 and Figure 12) show that the weaker and more ductile the aluminium grade, the better is the performance, in terms of PDE and RFd/P. For example, the energy dissipated by an auxetic core made by AL3 is nine times higher than AL2. Moreover, no energy dissipation noticed for AL1 as the latest is the high strength aluminium. The use of relatively weak grade, such as AL3 with yield point of 90 MPa, allows more deformation in the core and greater energy absorption. In return, RFd/P for AL3 was also less than that for AL1. Therefore, the aluminium grade AL3 (6063-T4) was selected for the UGAD due to its overall performance, low cost and high availability.

### 4.4. Cell Angle 

As mentioned earlier, the cell angle θ plays an important role in the performance of re-entrant auxetic structures, as it changes the Poisson’s ratio, auxetic behaviour and, consequently, PDE and RFd. In this section, three cell angles were considered, θ = 45°, 60° and 75°. Cell angles less than 45° were not taken, as interior cell surfaces may contact each other. The size of the auxetic core block was kept approximately as 200 × 200 × 200 mm. The exact total length, height, number of layers, and mass, of the three auxetic cores with three different cell angles are shown in Table 6. Other factors were kept constant such as the loading direction D1, cell dimension B (L = 10 mm), Grade AL3, t = 2.6 mm, t/L = 0.26, extrusion depth = 200 mm, and pulse load 500,000 N in 0.002 s.

**Figure 13:**
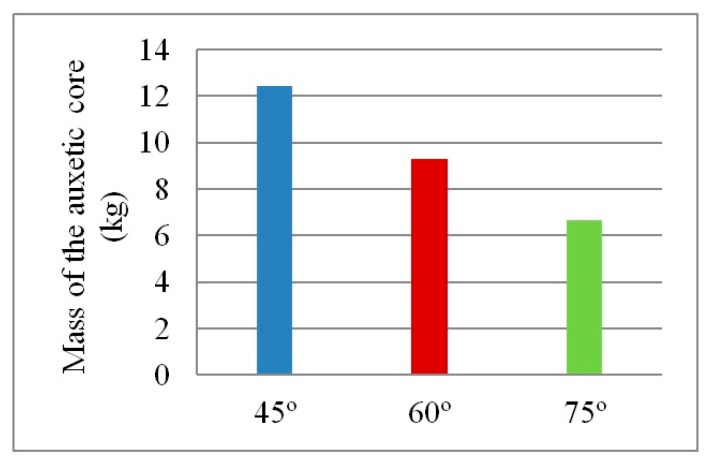
Mass of auxetic cores with 3 different cell angles, and L = 10 mm, t = 2.6 mm, t/L = 0.26.

**Figure 14:**
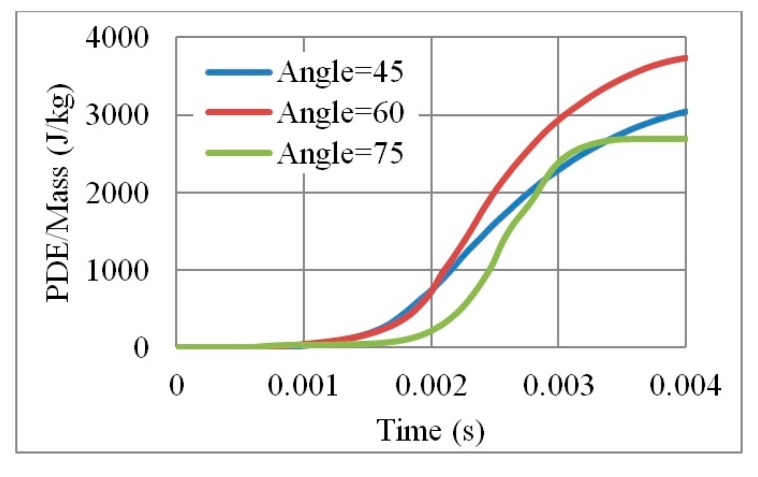
PDE/Mass with respect to time, for 3 different cell angles, of an auxetic core with L = 10 mm, t = 2.6 mm, t/L = 0.26.

Visibly, as the angle increases, the number of layers decreases, reducing PDE and the overall mass of the core. For instance, the mass of an auxetic core with θ = 45° is double that of θ = 75°, as illustrated in Figure 13. On the other hand, according to Imbalzano et al. [22]; reviewed in Table 3.2; the bigger the angle θ, the more energy dissipation is perceived. The contradiction in this physical behaviour is illustrated by the normalized PDE in Figure 14. Therefore, angle 60° showed to have the best PDE of 3700 (J/kg), as it had an average angle, number of layers and mass compared to other angles.

**Figure 15:**
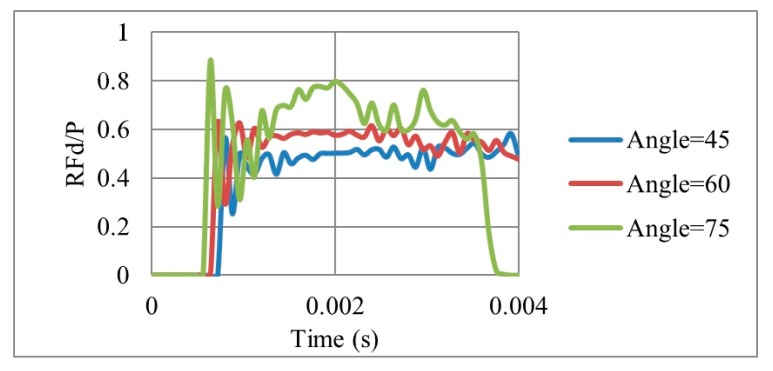
RFd/P with respect to time, for 3 different cell angles, of an auxetic core with L = 10 mm, t = 2.6 mm, t/L = 0.26.

**Figure 16:**
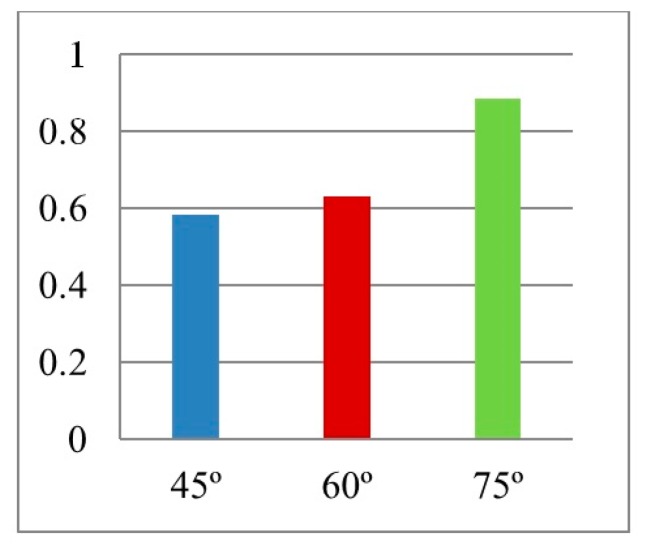
Max. value of RFd/P for 3 different cell angles.

Results for reaction forces (Figure 15) showed that the smaller the angle, the lower the reaction force. Peak values of RFd/P (Figure 16) for θ = 45°, 60° and 75° were 0.58, 0.62 and 0.88, respectively. The outcomes are consistent with other researchers’ conclusions [22]. However, as the peak RFd/P for θ = 45°, 60° are close to each other, θ = 60° had been selected for the UGAD as it had a clear higher PDE potential.

### 4.5. Number of Layers 

In addition to the previous parametric studies, the number of layers an auxetic core would need to absorb effectively an impact load also had to be checked. Here, three auxetic cores with three different numbers of layers were tested, which were four, eight and twelve layers. They have the same geometrical properties and loading conditions, loading direction D1, Grade AL3, Cell dimension B (L = 10 mm), t = 2.6 mm, t/L = 0.26, $\theta_{AXU}$ = 60°.

**Figure 17:**
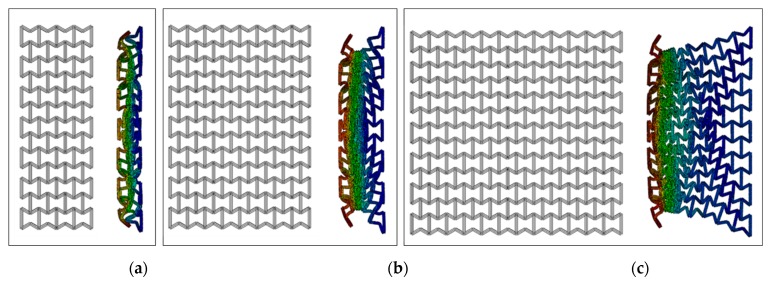
Deformation patterns of three auxetic cores with different numbers of layers of the same geometrical properties and loading conditions, having the same loading direction D1, Grade AL3, Cell dimension B (L= 10 mm), t = 2.6 mm, t/L= 0.26, $\theta_{Aux}$ = 60°, (**a**) four layers, (**b**) eight layers, (**c**) twelve layers.$\theta_{Aux}$.

Under the same impact load of 500,000 N in 0.002 s, the three auxetic cores responded differently, as shown in Figure 17. The four layers core was fully collapsed, while the eight and twelve layers were able to stop the impact before full densification is reached.

**Figure 18:**
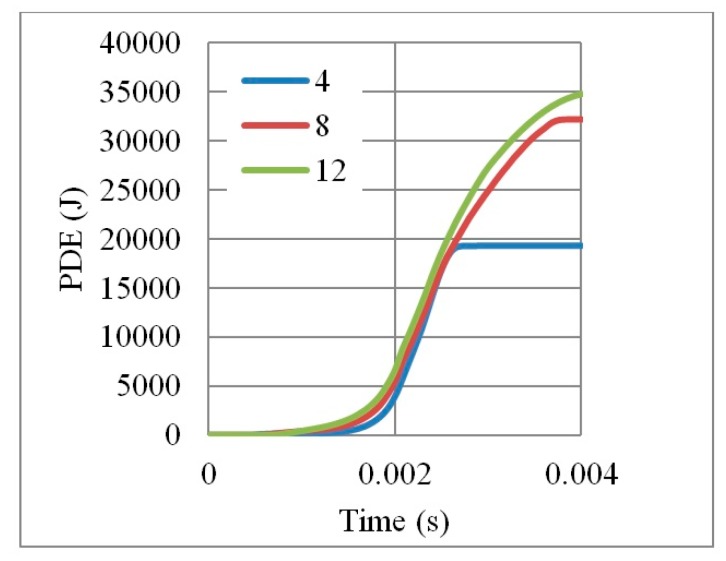
PDE with respect to time, for auxetic cores of different number of layers, having the same geometrical properties and loading conditions, L = 10 mm, t = 2.6 mm, t/L = 0.26, cell angle = 60°, AL3.

In terms of PDE (Figure 18), it can be seen that the more the number of layers, the more PDE is perceived due to the availability of more plastic hinges. These numerical findings match the results of Imbalzano et al. [22]. In addition, the change from four to eight layers raised the PDE dramatically by 74% (from 19,000 J to 33,000 J). In contrast, the PDE of twelve layers was only 6% higher than that of eight layers (from 33,000 J to 35,000 J). 

**Figure 19:**
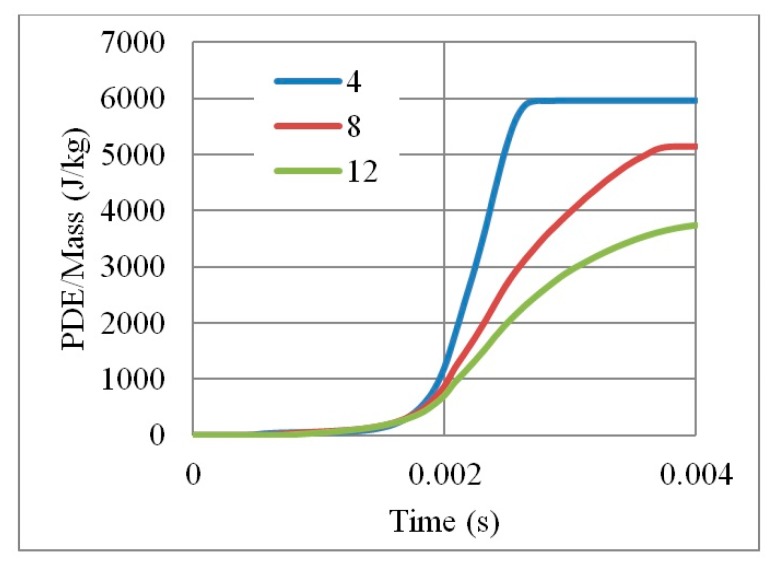
PDE/Mass with respect to time, for auxetic cores of different number of layers, having the same geometrical properties and loading conditions, L = 10 mm, t = 2.6 mm, t/L = 0.26, cell angle = 60°, AL3.

In the parametric study of optimum number of layers, normalizing the PDE by mass should not be considered as it misleads the physical interpretation. Figure 19 shows how PDE/Mass reversed the hierarchy (i.e., the four layers core seems to have the highest value of PDE because it was divided by the smallest mass).

**Figure 20:**
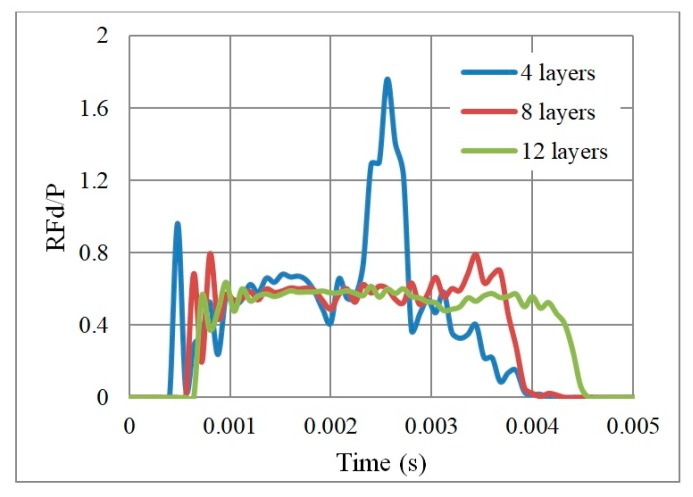
RFd/P with respect to time, of 3 auxetic cores with different number of layers of the same geometrical properties and loading conditions, having the same loading direction D1, Grade AL3, Cell dimension B (L = 10 mm), t = 2.6 mm, t/L = 0.26, $\theta_{Aux}\;$ = 60°.

**Figure 21:**
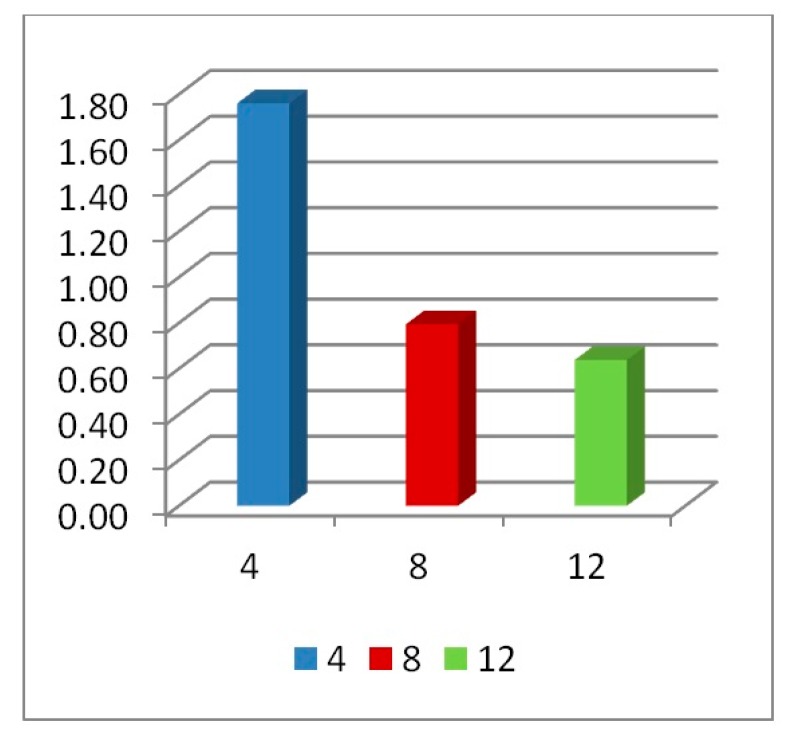
Peak value of RFd/P of 3 auxetic cores with different number of layers; having the same geometrical properties and loading conditions.

In terms of reaction forces (Figure 20 and Figure 21), the full collapse of the four layers led to a reaction on the support with 77% magnification of the applied load P (RFd/P = 1.77). On the other hand, the eight and twelve layers absorbed the impact transferring 79% and 64% of the applied load P, respectively. Zhang et al. [37], state that when the number of layers is greater than 10, the dynamic response of auxetic structures tends towards stability, i.e., less change in RFd/P and PDE should be expected (as seen in Figure 18 and Figure 20). 

### 4.6. Cell Wall Thickness t 

The parametric study, presented here, focused on six parameters that had to be modified for better performance of the uniaxial graded auxetic damper (UGAD). The selected parameters were the loading direction D1, cell dimension B (L = 10 mm), aluminium grade AL3 (6063-T4), cell angle θ = 60° and lastly; 8–12 layers were the range for effective number of layers. The cell wall thickness *t* is the only remaining parameter that has to be selected based on real loading from a structure subjected to blast or impact pressure.

**Figure 22:**
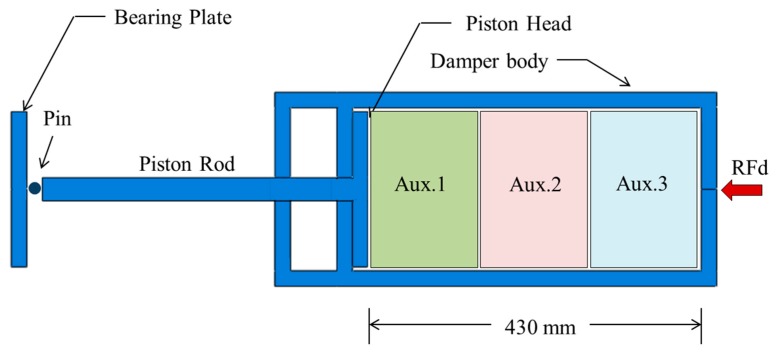
Uniaxial graded auxetic damper (UGAD) cross-section with three auxetic cores for three different blast levels.

As an example, a blast resistant gate can transfer the blast energy to a number of passive dampers. The latter, may absorb the energy, leading to less permanent deformations and less reaction forces. For more details on the way a blast resistant gate would distribute the blast to its supports (reaction forces), refer to Al-Rifaie and Sumelka [75]. Based on which, the cell wall thickness *t* of the auxetic core were selected for three levels of blast pressures, 3.3 MPa, 4.95 MPa and the maximum 6.6 MPa, achieved from 50 kg, 75 kg and 100 kg of TNT at 5 m stand-off distance (R), respectively. Therefore, three auxetic cores with three different values of *t* would be placed in the damper body as a “*graded auxetic system*” with graded properties described using step functions, as shown in Figure 22. The values of the selected cell wall thickness for the three auxetic cores are listed in Table 7. Each auxetic core has eight layers leading to a total 24 layers that can fit into the damper body (430 mm). 

## 5. Final Properties of the UGAD 

Based on the parametric studies conducted in Section 4, the final geometrical and mechanical properties of the three auxetic cores are described here in this section. Table 7 shows the auxetic cores and lists their properties. They have the same L, θ, material grade, size and hence, overall volume. The cell-wall thickness ***t*** is the variable parameter; which in return; leads to the distinct mass, density and relative density. The density of each auxetic core (ρ) was achieved from dividing the mass of each core by the undeformed volume V (V = 140 × 200 × 200 mm = 5.6 × 10^6^ mm^3^). The relative density $\rho^{*}$ is the ratio of the auxetic core density (ρ) to the density of the material used ($\rho_{s}$):

(5) $$\rho^{*}=\rho /\rho_{s}$$

The relative density $\rho^{*}$ can also be calculated analytically using [11]:

(6) $$\rho^{*}=\frac{\rho }{\rho_{s}}=\frac{1}{2}\;\frac{t}{L}\;\frac{\left(\frac{L_{1}}{L}+2\right)}{\cos\theta \left(\frac{L_{1}}{L}+\sin\theta \right)}$$

The relative density is an important parameter as it shows also the void ratio in cellular metals. The void ratio can be calculated as:

(7) $$\frac{V_{v}}{V}=100(1-\rho^{*})$$

It can be perceived from Table 7 that the relative density increased with the increasing *t*. In addition, Aux. 1 had the highest void ratio of 77.7% compared to Aux. 3 that had 65%. 

**Figure 23:**
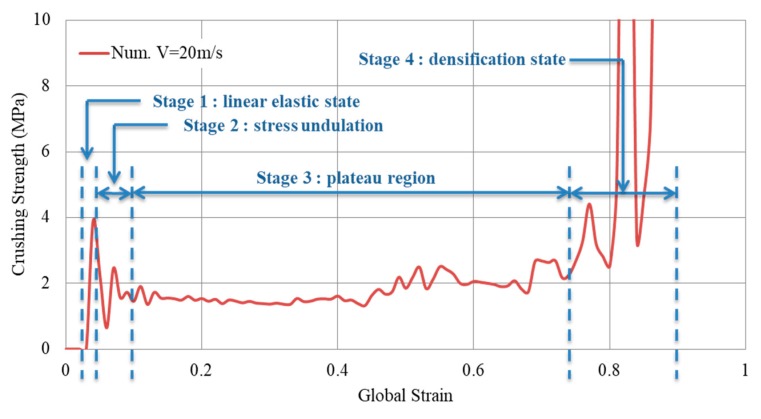
Stress-strain curve of Aux. 1 under 20 m/s impact velocity, showing the four stages of crushing re-entrant auxetics.

Crushing a cellular structure pass through four states. The 1st is the linear elastic state (cell wall bending). The 2nd is the stress undulation (cell wall collapse). The 3rd is the plateau region where plastic bending occurs. The last is the densification state, when the cell walls touch each other [21]. It is the plateau region that is important in characterizing the dynamic crushing of auxetic structures for energy absorbing applications [37]. The plateau stress can be defined as “*the average nominal stress between the first stress peak and the compressive stress corresponding to the densification strain*” [37]. Figure 23 shows the stress-strain curve of Aux. 1 under 20 m/s constant impact velocity, highlighting the four stages of crushing a reentrant auxetic structure mentioned above. The crushing strength was calculated based on the RFd divided by the impact area of 40,000 mm^2^(200 × 200 mm). 

Based on the rigid, perfectly plastic, locking material simplified model (r-p-p-l model), the theoretical expression for the dynamic plateau stress (dynamic crushing strength) of re-entrant auxetics is [21,76]:

(8) $$\sigma_{d}=\sigma_{0}+\frac{\rho }{\varepsilon_{d}}\;v^{2}$$

where, ν is the impact velocity, $\varepsilon_{d}$ is the locking strain, which can be found from the stress-strain curve under the quasi-static uniaxial compression, $\sigma_{0}$ is the static plateau stress calculated following Gibson and Ashby [11] as:

(9) $$\sigma_{0}=\frac{2\;\sigma_{ys}\;{\left(\frac{t}{L}\right)}^{2}}{3}$$

**Figure 24:**
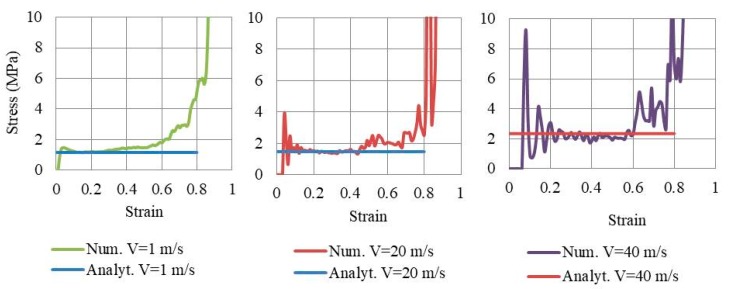
Numerical stress-strain curve of Aux. 1 under different impact velocities, compared to the analytical “dynamic crushing strength”.

Implementing the periodic collapse mechanism of re-entrant auxetics, Hou et al. [21] derived the analytical expression of “dynamic crushing strength” as a function of the cell-wall aspect ratio ***t****/L* and the impact velocity ν:

(10) $$\sigma_{d}=\left[\frac{2\;\sigma_{ys}\;{\left(\frac{t}{L}\right)}^{2}}{3}\right]+\left[\frac{16\;\rho_{s}\left(\frac{t}{L}\right)}{7\;\sqrt{3}-28\;\left(\frac{t}{L}\right)}\right]\;v^{2}$$

where the first part is the static plateau stress (Equation (9)) and the second part is the additional hardening (based on impact velocity). By substituting the values of t, L, $\sigma_{ys}$ and $\rho_{s}$ in Equation (10); for each auxetic core (Aux. 1, Aux. 2 or Aux. 3); the dynamic crushing strength can be found analytically for any impact velocity ν. It is important to highlight that the second part of Equation (10) approaches to zero when the impact velocity is less than or equal to 1 m/s (i.e., equal to static plateau stress). Therefore, three velocities were selected to compare the analytical and numerical dynamic crushing strength, namely, 1 m/s, 20 m/s and 40 m/s. The analytical solution of Equation (10) for Aux. 1, for example, gives the plateau stress of 1.17 MPa, 1.47 MPa and 2.35 MPa for the three velocities, respectively. Figure 24 shows the numerical stress-strain curve of Aux. 1 under different impact velocities, compared to the analytical “dynamic crushing strength”. A very good agreement can be seen in the plateau region for all impact velocities. The comparison of analytical and numerical outcomes presented here can be considered as a validation of the auxetic core numerical model. 

**Figure 25:**
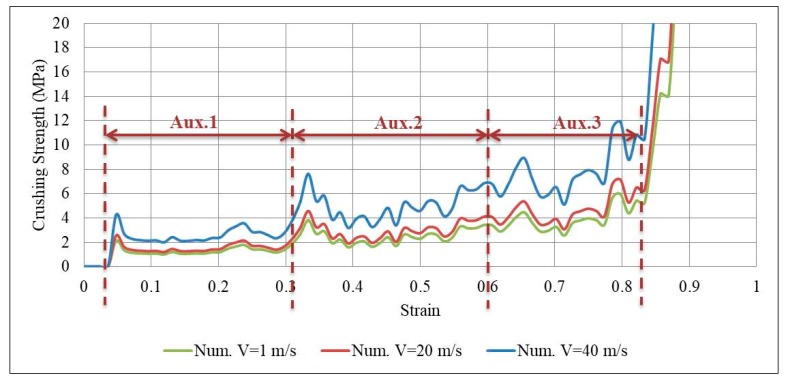
Numerical stress-strain curve of the three auxetic cores together in the UGAD under different impact velocities, 1 m/s, 20 m/s and 40 m/s.

The stress-strain curve of the three auxetic cores together in the UGAD under different impact velocities; 1 m/s, 20 m/s and 40 m/s; are shown in Figure 25. The progressive collapse is evident, through compressing Aux. 1, then Aux. 2 and Aux. 3, in sequence. The performance of the three auxetic cores together have led to a wide plateau region (80% of total crushing strain) and variant strength range (1–10 MPa), which in return, can justify the superior performance of the UGAD under different blast levels.

## 6. UGAD Applications 

**Figure 26:**
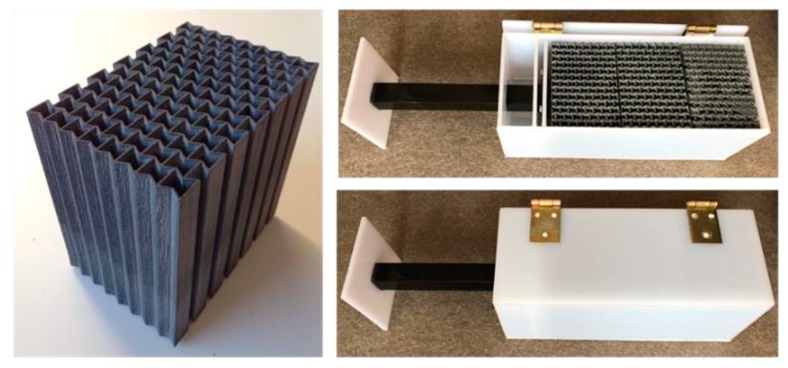
A three dimensional (3D) printed prototype of the UGAD.

The novel uniaxial graded auxetic damper (UGAD) proposed in this research can be designed to withstand different impact or blast pressures, based on changing the cell-wall thickness of its auxetic cores and even number of cores in the damper body. The UGAD can be used in different scales for different structural applications, such as; blast resistant doors/gates; blast-resistant façade for retrofitting sensitive buildings; elevator (absorbing unexpected crash of elevators or cable failure in multi-story buildings); crash energy absorbing systems in motor vehicles front bumpers; and many other possible applications. Figure 26 shows a 3D printed prototype of the UGAD suggested in this research. The prototype has different materials, and hence can not be used for experimental testing/validation.

The limitation of this proposed UGAD is the lack of full scale experimental validation that is the aim of the authors for future research-nonetheless the presented virtual designing bases on the comprehensive experimental study in [72,73,74]. In addition, the production of “aluminium” cores requires slow and costly metal 3D printing. However, this problem can be solved using the pre-prepared “extrusion mold” for fast large-quantity production. 

## 7. Conclusions 

A detailed parametric study was conducted to design a new uniaxial graded auxetic damper (UGAD). Then, its static and dynamic constitutive relations were derived and validated analytically. The proposed UGAD consists of four main components, which are the bearing plate, piston, damper body and three auxetic cores, for three different blast/impact levels. The parametric study, focused on six parameters that had to be optimized for better performance of the UGAD. The selected parameters were the loading direction D1, cell dimension B (L = 10 mm), aluminum grade AL3 (6063-T4), cell angle θ = 60° and lastly; 8–12 layers was the range for the effective number of layers. In terms of the cell wall thickness of the auxetic cores, the lightest-most effective three auxetic cores that was fitted in the UGAD; namely Aux. 1, Aux. 2 and Aux. 3, had the cell wall thickness ***t*** of 1.4 mm, 1.8 mm and 2.2 mm, respectively. The selection of optimum parameters was based on the ratio of the reaction force to the applied load (RFd/P) and plastic dissipation energy (PDE). A very good agreement was noticed between the numerical and analytical plateau region for all impact velocities, which can be considered as a validation of the auxetic core numerical model. The performance of the three auxetic cores together have led to a wide plateau region (80% of total crushing strain) and variant strength range (1–10 MPa), which in return, can justify the superior performance of the UGAD under different blast levels. The auxetic nature (negative Poisson’s ratio = −0.3) and transverse shrink make it easier to change the compressed auxetic core in the UGAD after a blast event. 

The new proposed UGAD can be used in different scales for different structural applications, such as blast-resistant façade and crash absorbers in the automotive industry. For future research, authors recommend trying different auxetic topologies, such as the recently introduced re-entrant star-shaped honeycomb (RSH) [77] and star-arrowhead honeycomb (SAH), [78]. Herein, the additive manufacturing techniques are of main importance. 

## Figures and Tables

**Table 1:** Fixed and variable geometrical parameters of the UGAD auxetic core.

Fixed Parameters		Variable Parameters
UGAD chamber internal space 210 × 210 × 430 mm	dimensions:	Cell dimensions L_1_, L_2_, L and H while L_1_ = 2 L
Auxetic core extrusion depth = 200 mm		Cell wall thickness *t*
Auxetic core height = 200–210 mm		Cell angle
Cell wall aspect ratio = t/L = 0.10, 0.15, 0.20		Number of layers

**Table 2:** The three aluminium grades used for the auxetic core and their applications.

Symbol	AL Grade	Strength	Yield Point (MPa)	Applications
AL 1	7075-T6	High	546	Aerospace and defence
AL 2	6061-T6	Medium	324	General Structural Applications
AL 3	6063-T4	Low	90	Door, windows, furniture

**Table 3:** Material parameters of the three aluminium grades used in the UGAD auxetic core.

	Description	Unit	AL7075−T6 [72]	AL6061−T6 [73]	AL6063−T4 [74]
E	Modulus of Elasticity	MPa	71.7 × 10^3^	69 × 10^3^	68.9 × 10^3^
ν	Poisson’s ratio	−	0.33	0.33	0.33
ρ	Mass density	t/mm^3^	2.81 × 10^−9^	2.703 × 10^−9^	2.703 × 10^−9^
A	Yield Strength	MPa	546	324	89.6
B	Ultimate Strength	MPa	678	113	172
n	Work−hardening exponent	−	0.71	0.42	0.42
$\dot{\varepsilon_{0}}$	Reference Strain rate	s^−1^	1 × 10^−4^	1 × 10^−4^	1 × 10^−4^
C	Strain rate factor	−	0.024	0.002	0.002
$D_{c}$	Critical Damage	−	0.3	0.3	0.3
$p_{d}$	Damage threshold	−	0	0	0
$c_{p}$	Specific heat	mm^2^ k/s^2^	960 × 10^6^	910 × 10^6^	910 × 10^6^
χ	Inelastic heat fraction	−	0.9	0.9	0.9
$T_{m}$	Melting Temperature	k	750	925	616
$T_{0}$	Room Temperature	k	293	293.2	293.2
m	Thermal−softening exponent	−	1.56	1.34	1.34
$d_{1}$	−	−	−0.068	−0.77	−0.77
$d_{2}$	−	−	0.451	1.45	1.45
$d_{3}$	−	−	−0.952	0.47	0.47
$d_{4}$	−	−	−0.036	0.00314	0.00314
$d_{5}$	−	−	0.697	1.6	1.6

**Table 4:** Loading directions D1 and D2 and their effect on the collapse mode and deformation of an auxetic core (t = 0.75 mm, L = 5 mm, t/L = 0.15, θ = 60°, AL2 grade).

Time (s)	Direction D1 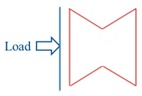	Direction D2 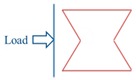
0	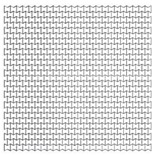	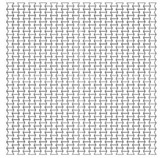
0.001	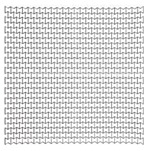	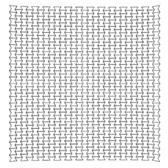
0.002	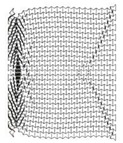	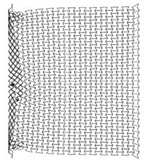
0.003	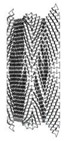	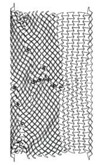
0.004	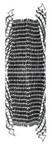	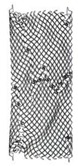

**Table 5:** Auxetic cores with three different cell dimensions and their properties.

	A	B	C
Shape	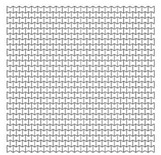	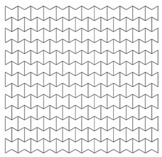	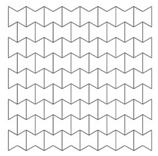
L	5	10	15
*t*	1	2	3
Total Length	208	208	208
Total Height	205	200	210
No. of Layers	24 × 27	12 × 13	8 × 9
Mass (kg)	7.212	7.158	7.639
Fixed Factors	θ = 60°, t/L = 0.2, Extrusion depth = 200 mm, pulse load 500,000 N in 0.002 s		

**Table 6:** Auxetic cores with three different cell angles and their properties.

	Angle = 45°	Angle = 60°	Angle = 75°
Shape	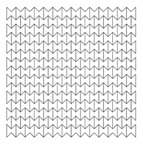	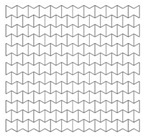	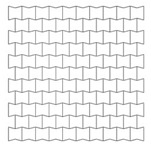
Total Length	198	208	193
Total Height	201	200	194
No. of Layers	14 × 15	12 × 13	10 × 11
Mass (kg)	12.4	9.3	6.6
Fixed Parameters	Loading direction D1, Cell dimension B (L = 10 mm), Grade AL3, t = 2.6 mm, t/L = 0.26, Extrusion depth = 200 mm, pulse load 500,000 N in 0.002 s		

**Table 7:** The three auxetic cores with their geometric and mechanical properties.

	Aux.1	Aux.2	Aux.3
Shape	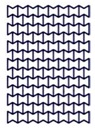	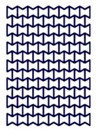	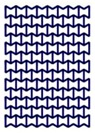
Shared Parameters	L = 10 mm, cell angle θ = 60°, Grade AL3 ($\rho_{s}\;$= 2.703 × $10^{-9}$ t/mm^3^), Size = 140 × 200 × 200 mm, volume of one core V = 5.6 × 10^6^ mm^3^		
t(mm)	1.4	1.8	2.2
t/L	0.14	0.18	0.22
Mass (ton)	0.00338	0.00434	0.00530
Mass (kg)	3.38	4.34	5.30
Density ρ (t/mm^3^)	6.036 × 10^−10^	7.75 × 10^−10^	9.46 × 10^−10^
Relative Density $\rho^{*}=\rho /\rho_{s}$	0.223	0.287	0.35
Void Ratio %	77.7	71.3	65
